# Most oncological pancreas resections must consider the mesopancreas

**DOI:** 10.1186/s12885-025-13599-x

**Published:** 2025-02-04

**Authors:** S. A. Safi, S. David, L. Haeberle, S. Vaghiri, T. Luedde, C. Roderburg, I. Esposito, G. Fluegen, W. T. Knoefel

**Affiliations:** 1 Department of Surgery (A), Heinrich-Heine-University and University Hospital Duesseldorf, Moorenstr. 5, 40225 Duesseldorf, Germany; 2 Institute of Pathology, Heinrich-Heine-University and University Hospital Duesseldorf, Moorenstr. 5, 40225 Duesseldorf, Germany; 3 Department of Gastroenterology, Hepatology and Infectious Diseases, Heinrich-Heine-University and University Hospital Duesseldorf, Moorenstr. 5, 40225 Duesseldorf, Germany

**Keywords:** PDAC, Ductal adenocarcinoma of the pancreas, Pancreatic cancer, CRM, Mesopancreatic excision, Mesopancreas

## Abstract

**Background:**

In preoperative staging for patients with a ductal adenocarcinoma of the pancreatic head (PDAC), resectability is anatomically characterized by the possible clearance of the medial vascular grove. Borderline resectable PDAC patients who retain an increased risk of infiltration to the portomesenteric system and/or arterial vasculate are candidates for neoadjuvant therapy. However, redefined pathological analysis revealed the dorsal resection margin to be similar at risk for R1 resection. Mesopancreatic excision (MPE) aims to secure the integrity of the dorsal and ventral resection margins. The existence of the mesopancreas (MP) is inevitable, since the pancreas is of a secondary retroperitoneal nature and the dorsal as well as ventral fascial coverings define the peripancreatic compartment anatomy. It remains unknown if the MP area is only infiltrated in high-risk PDAC patients or if MPE during pancreatoduodenectomy should be employed for localized PDAC patients as well.

**Methods:**

Patients who underwent upfront pancreatoduodenectomy were included. CRM evaluation and analysis of the MP was standardized in all patients. Patients were sub-grouped by the infiltration status of the vascular groove (localized disease: LOC). In LOC patients there was evidently no cancerous infiltration into the medial vascular groove (true + primary resectable).

**Results:**

Two hundred eighty-four consecutive patients who underwent pancreatoduodenectomy were included (169 LOC patients). In LOC patients the MP infiltration rate remained high but was significantly lower when compared to advanced PDAC patients (MP + 69.2% vs. 83.5%, *p* = *0.005*). In LOC patients, CRM resection status of the dorsal resection status remained significantly affected by the MP infiltration status (R0CRM– 80.5% vs. 62.8%, *p* = *0.019*).

**Conclusion:**

These important findings clearly show underestimated tumor extensions into the mesopancreas even in localized, primary resectable PDAC patients who are currently amenable for upfront resection. Synergistically to total mesorectal or mesocolic excision, which is applied to all stages of colorectal disease, MPE is justified in primary resectable patients as well. Therefore, MPE should be employed in all PDAC patients. Since the infiltration status of the mesopancreas was a significant factor for incomplete resection in primary resectable PDAC patients, neoadjuvant treatment options for must be discussed.

## Introduction

The implementation of the circumferential resection margin (CRM) and the recommendations of the Royal College of Pathologists has resulted in a better understanding of true tumor extensions and superior survival stratification in PDAC patients [1, 2]. The modified pathological evaluation includes a separate investigation of the ventral and dorsal pancreatic surfaces, as well as the medial vascular groove (i.e. the groove of the superior mesenteric vein and the superior mesenteric artery) by a separate inking procedure [3, 4]. The medial and dorsal resection margins remained the major sites for insufficient margin clearances [5].

This low rate of true margin negative resections (R0CRM–) is either a result of insufficient preoperative stratification of patients or an insufficient degree of surgical resection [6–10]. On the other hand, the implementation of the compartment anatomy in the colorectal system, complete mesocolic and total mesorectal excision, redefined the contemporary surgical understanding of resection margins by utilizing embryologic derived anatomical boundaries [11, 12]. This change in surgical perspectives resulted from an anatomical and embryologic understanding of a secondary retroperitoneal nature of these organs with the existence of fusion fascia to be utilized as resection margins. Since the pancreas is located secondarily retroperitoneal as well [13, 14], the existence of a mesopancreas should not be surprising and the oncological outcome and relevance of the mesopancreas in PDAC patients was already studied by us and others [15–21].

The mesopancreas represents embryologically the ventral and dorsal resection margin, which next to the medial resection margin, is a major sight for incomplete tumor clearance [18]. Patients are currently stratified into primary-, borderline- and non-resectable solely according to the presumed radiographic infiltration status of the vascular system located at the medial resection margin (vascular groove) [6], radiological findings however do not give us a definitive certainty until a resection and histopathological findings proof otherwise. It is unknown if in PDAC patients who were preoperatively correctly staged primary resectable, i.e. in whom postoperatively the PDAC remained localized (true positive primary resectable), the mesopancreas is infiltrated or not.

The aim of this study is to provide evidence of the mesopancreatic fat infiltration status in PDAC patients who have been histopathological stratified according to tumor extensions into the medial vascular groove [6]. We hypothesized to improve local tumor [22], the analysis of the mesopancreas in a thoroughly stratified patient cohort could result in a better understanding of these delicate tumor extensions. It remains unknown whether mesopancreatic excision during pancreatoduodenectomy should be reserved for patients with an infiltrated vascular groove (i.e., borderline resectable/advanced stage of disease), or if this technique secures tumor margins in in localized PDAC patients as well.

## Material and method

### Patient selection and demographic data

This analysis included patients who had undergone pancreatoduodenectomy (PD) for pancreatic ductal adenocarcinoma (PDAC) at the University Hospital of Duesseldorf between 2015 and 2022. Patients were selected from a prospectively maintained database, without restriction on tumor stage or microscopic margin status. Only M0 resected patients were included. Inclusion criteria were confined to individuals with PDACs of the pancreatic head, who had not received neoadjuvant chemotherapy, and for whom the histopathological evaluation of the circumferential resection margin (CRM) was primarily available [2, 3, 23]. Excluded from the study were palliative patients or patients undergoing surgery for periampullary neoplasms or for resections of the distal pancreas. Data on TNM classification, tumor grade, perineural involvement, as well as lymphatic and venous invasion, were sourced from the primary pathology documents. The staging system followed the 8th edition of the UICC TNM classification.

**Fig. 1:**
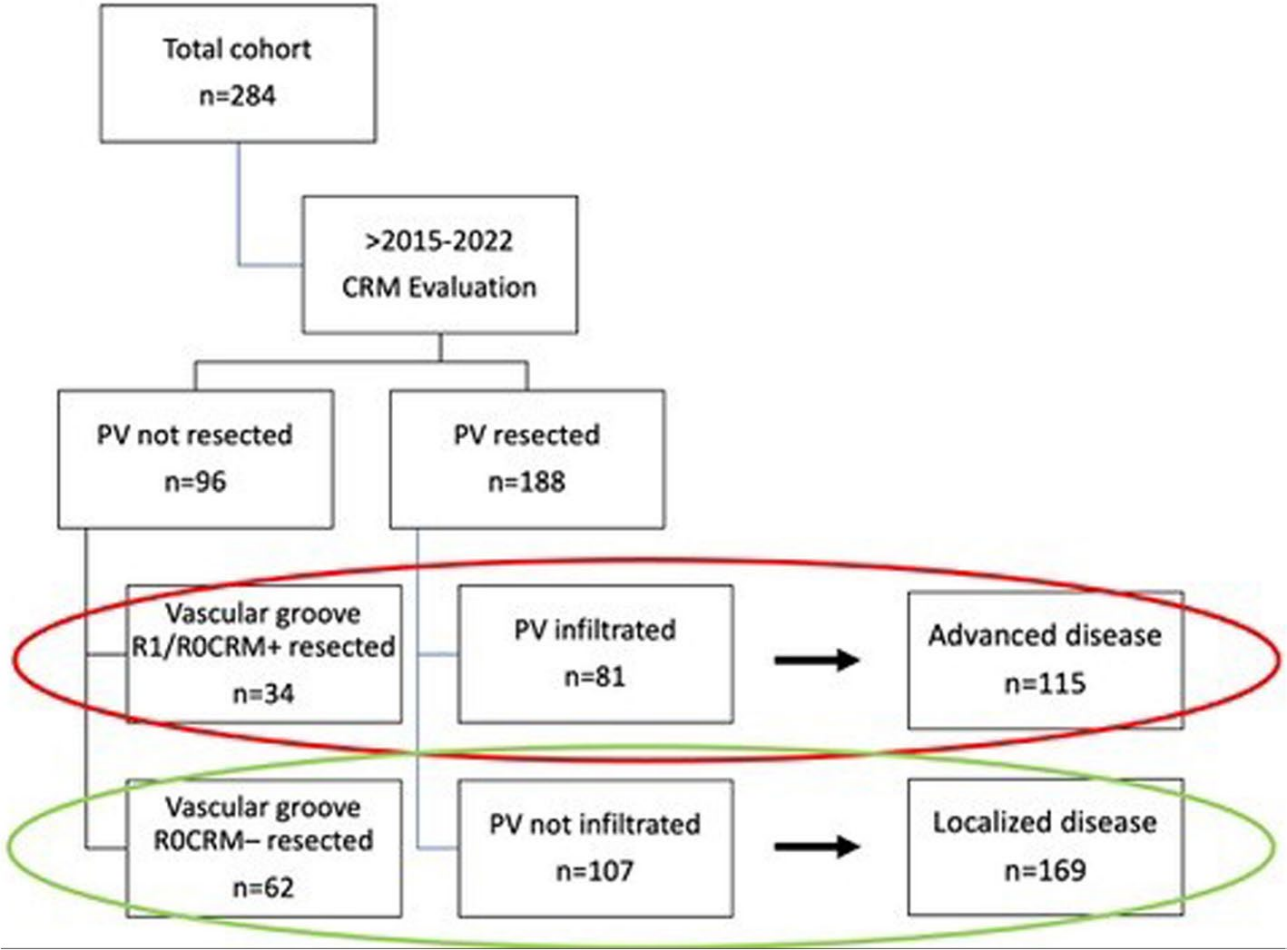
Flow chart of patient work-up. (CRM = circumferential resection margin)**.** Patients were postoperatively stratified according to tumor extensions by true infiltration status of the vascular groove. Localized disease = LOC, patients with no vascular involvement and/or R0CRM– resection at the medial vascular groove. Advanced disease = ADV, patients with vascular involvement and/or R1/R0CRM + resection at the medial vascular groove

**Fig. 2:**
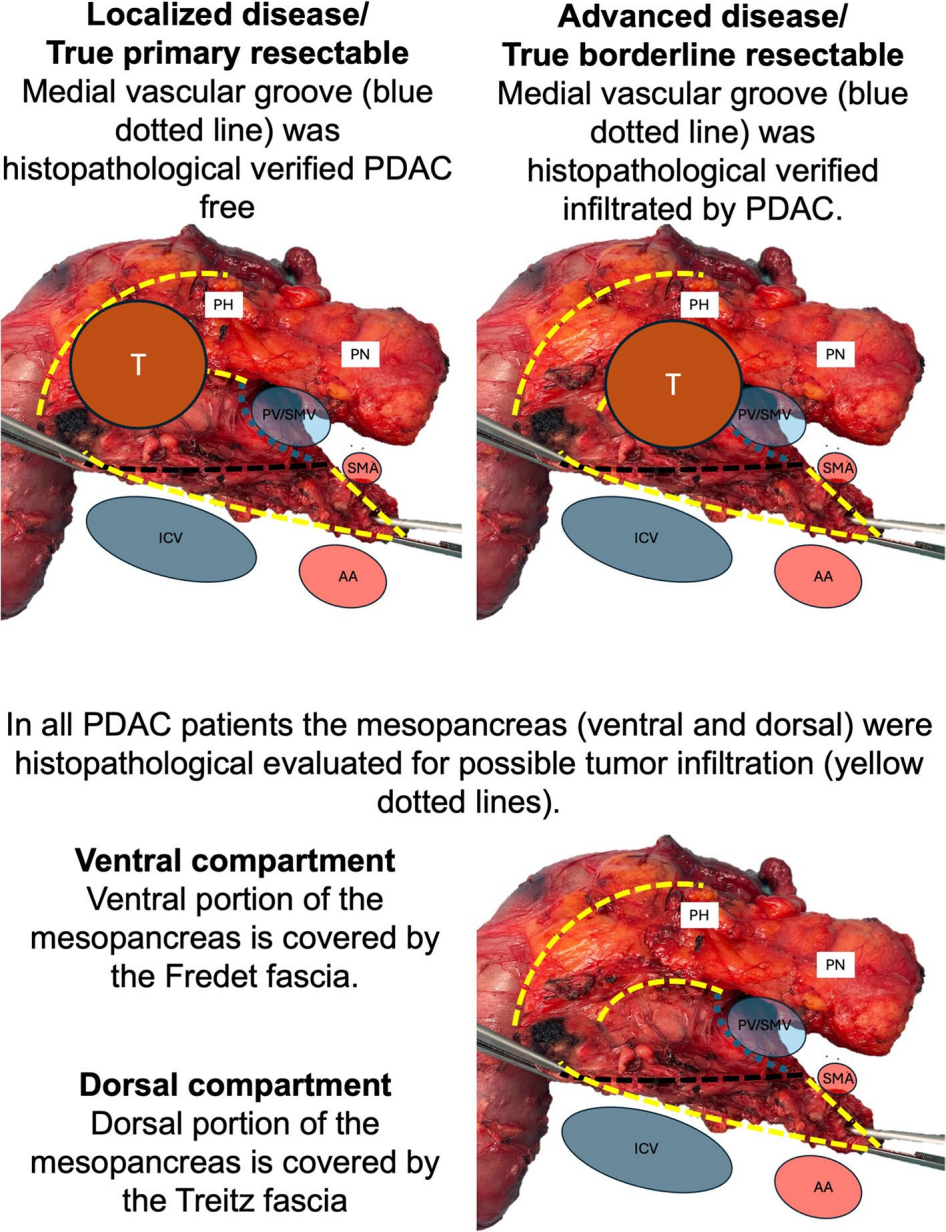
Visualized flow chart of localized (LOC) and advanced (ADV) PDAC patients. Through the implemented CRM we assessed the tumor extensions into the medial vascular groove (blue dashed line). This area is of primary concern during resectability assessment (**A** and **B**). The black dashed line shows the border between the pancreatic parenchyma and dorsal part of the mesopancreas. In both groups the infiltration status of the mesopancreas was evaluated (tissue beyond yellow dashed lines at the ventral and dorsal section) (**C** and **D**). (AA: abdominal aorta, ICV: inferior caval vein, PH: pancreatic head, PN: pancreatic neck, PV: portal vein, SMA: superior mesenteric artery, T: tumor, UP: uncinate process)

The study cohort was divided into two groups by the histopathological evaluation of the vascular grove: Localized PDAC Patients (Group LOC) underwent PD with or without vascular resection and had no histological infiltration of the vessels or were histologically R0CRM– resected (no vascular resection) at the medial resection margin (Figs. 1 and 2). Thus, in the localized PDAC group the vascular groove had a R0CRM– resection status. We hypothesized that localized PDAC patients had no risk for an R1 resection at the medial resection margin (primary resectable), leaving the dorsal resection [5] only at risk (mesopancreas).

Patients who underwent PD with vascular resection and showed a positive infiltration status of the vessels (portal vein and/or superior mesenteric vein) or received PD without vascular resection but had an infiltration of the medial vascular groove or tumor growth extending outside the pancreas (R1/R0CRM +), were defined as advanced disease (ADV) (Figs. 1 and 2). Consequently, advanced PDAC patients without vascular resection had tumor infiltration into the vascular groove (R1 resection status) or if vascular resection was performed showed a positive infiltration into the PV (independent on resection status). Since we produced a confounder in both resection rate and vascular status, analysis of the R-status was not performed because of known bias. We only utilized this group to identify PDAC patients with a localized disease (Fig. 1).

### Operative procedure

The surgical approach by mesopancreatic excision was already thoroughly described by Safi et. al. both for pancreatic head malignancies and malignancies of the distal pancreas [18, 24] (Fig. 2). In summary, the aim of the procedure is a circumferential dissection of the peripancreatic tissue and structures integrating the peripancreatic facial system as anatomical landmarks. The aim of the procedure is to preserve the fascial sheets [23, 25] and their integrity during dissection bevor a point of no return is reached [18]. Surgical dissection and mobilization of the dorsal plain is followed by the fascia of Treitz during Kocher Maneuver [25]. The fascial integrity of the ventral aspect of the mesopancreas is achieved during mobilization of the right colonic flexure; dissections are carried out in vicinity of the mesocolon to preserve the duplication fascia of Fredet on the ventral mesopancreas [23].

### Statistics

Statistical analysis of clinico-pathological data was conducted using the Wilcoxon signed-rank test. The Mann–Whitney U test was employed to assess numerical variables and examine associations between clinico-pathological factors. Categorical variables were analyzed using either the chi-square test or Fisher's exact test, as appropriate. Univariate survival analysis was performed by the log-Rank test. All statistical computations were performed with SPSS for Windows (version 26.0; SPSS, Inc., Chicago, IL, USA). A *p*-value of less than 0.05 was considered statistically significant.

## Results

### Demographic data

Figures 1 and 2 visualize our workflow and subgroup stratification. From the total study cohort of 284 patients, 188 patients (66.2% of the total cohort) received a simultaneous resection (tangential or segmental) of the PV/SMV during pancreatoduodenectomy because of presumed infiltration. Of these 188 patients, 81 had a histologically verified vascular infiltration (48.1%; true positive), while in the remaining 107 patients the vessels were not histologically infiltrated (56.9%; false positive) (Fig. 1). In the remaining 96 patients (33.8% of the total cohort), PD was performed without vascular resection (preoperatively presumed to be localized PDAC) and the infiltration status of the vascular groove was utilized for stratification. In 62 out of the 96 patients (64.5%; true negative) margin clearance was successfully achieved without vascular resection, whereas in the remaining 34 patients (35.4%; false negative) resection status was insufficient (R1/R0CRM +), and a vascular resection would have been necessary. We thereby identified 169 PDAC patients with a localized, primary-resectable disease. In these patients the medical vascular groove was evidently cancer free (Fig. 1).

Patients have therefore been sub-grouped as followed: (1) Local disease (LOC): patients with simultaneous vascular resection but without cancerous infiltration of the vessel (false positive) and patients without vascular resection and with successful margin clearance (true negative). (2) Locally advanced disease (ADV): patients with simultaneous vascular resection and cancerous infiltration of the vessel (true positive) and patients without vascular resection and insufficient margin clearance at the vascular groove (false negative) (Figs. 1 and 2).

Table 1 summarizes clinico-pathological characteristics of the total study cohort stratified according to the current radiographic resectability criteria. In all 284 patients, CRM was implemented as previously described [7] The median age of all patients at the time of surgery was 69.5 years (range 17.0–90.0 years).

**Table 1:** Demographic data of patients stratified according to tumor extensions into the vascular groove. Statistical significance was calculated by chi squared test and Wilcoxon test. ** indicates a *p-value* ≤ *0.01*; * indicates a *p-value* ≤ *0.05*

	**Localized disease (LOC)** *n* = 169		**Locally advanced disease (ADV)** *n* = 115		***p-value***
**Age in years** (median (range))	69 (17–90)		70 (34–87)		*1.000*
**Sex**	**n**	**%**	**n**	**%**	*0.261*
Female	74	43.7	45	39.1	
Male	95	56.3	70	60.9	
**T-Status**					
T1	14	8.3	2	1.7	0.010**
T2	85	50.3	55	47.8	
T3	67	39.6	52	45.2	
T4	3	1.8	6	5.2	
**N-Status**					0.012*
N0	44	26.0	14	12.2	
N1/N2	125	74.0	101	87.8	
**G-Status**					*0.422*
G2	96	56.8	63	54.8	
G3	73	43.2	52	45.2	
**Pn-Status**					0.018*
Pn0	42	24.9	15	13.1	
Pn1	127	75.1	100	86.9	
**V-Status**					< 0.001**
V0	135	79.8	63	54.8	
V1	34	20.1	52	45.2	
**L-Status**					0.003**
L0	95	56.2	45	39.1	
L1	74	43.8	70	60.9	

### UICC 8th edition staging variables stratified according to resectability

When evaluating common relevant staging variables, ADV patients were significantly more likely to suffer from advanced stages of disease (higher T- and N-stage; positive Pn-, V- and L-status) (Table 1). Of the ADV patients, 50.4% had a PDAC ≥ T3, whereas only 41.4% of the LOC patients suffered from an advanced (≥ T3) T-stage (*p* = *0.010*). Of the extended staging variables (Pn, L and V), the percentile difference was the greatest in the microscopic vascular status (V): whereas microscopic vascular infiltration was evident in 45.2% of ADV patients, this was observed in only 20.1% of the LOC patients (*p* < *0.001*) (Table 1).

### Mesopancreas and resection status

In 213 patients (75%) tumor cells were detected in the mesopancreas (Table 2). When the infiltration status of the mesopancreas was stratified according to the tumor extensions into the vascular groove, LOC patients were significantly less prone to harbor tumor cells in the MP (MP + : 69.2% vs. 83.5%) (*p* = *0.005*) (Table 2).

**Table 2:** Mesopancreatic fat infiltration and resection status of patients stratified according to tumor extension status of the vascular groove. MP infiltration in localized PDAC patients remained with 69% high. R-status of the dorsal resection margin was significantly affected by MP infiltration status. Statistical significance was calculated by chi squared test. ** indicates a *p-value* ≤ *0.01*

	**Localized disease (LOC)** *n* = 169		**Locally advanced (ADV)** *n* = 115		***p***-value
**Mesopancreas**	**n**	**%**	**n**	**%**	0.005**
Infiltration +	117	69.2	96	83.5	
Infiltration –	52	30.8	19	16.5	
				
	**Resection status dorsal** **(LOC patients)**				
	R0CRM– n = 113		***p-value***		
**Mesopancreas**					
Infiltration +	*n* = 73 62.8% out of MP + LOC patients		***0.019***		
Infiltration –	*n* = 40 80.5% out of MP– LOC patients				

Interestingly, in LOC patients, MP infiltration status was a factor indicative of incomplete resection at the dorsal resection margin (Table 2). In LOC patients with MP + infiltration, R0CRM– resections were achieved in only 62.8%; whereas in patients without MP infiltration, R0CRM– resections were achieved in over 80.5% (*p* = *0.019*) (Table 2, bottom left). Thus, current stratification models for resectability (medial vascular groove) only partially reflect true tumor extensions, since resection status was significantly affected again by the infiltration status of the mesopancreas.

### Survival analysis

**Fig. 3:**
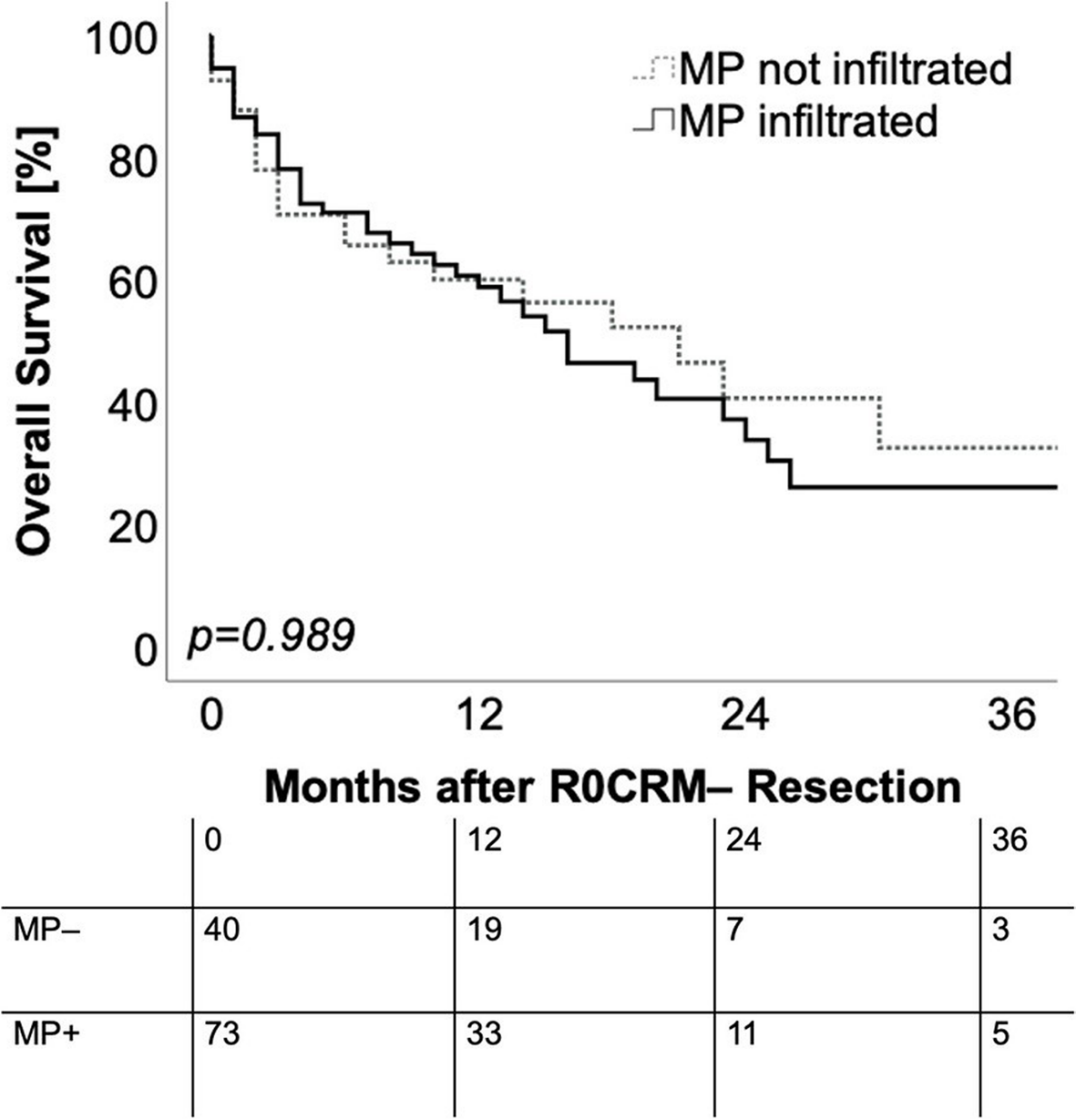
Kaplan–Meier curves for overall survival in correlation with positive and negative MP infiltration status in R0CRM– resected localized PDAC patients. The prognostic effect of R0CRM– resection was not influenced by mesopancreatic fat infiltration. *MP* = *mesopancreatic.* Log rank test was used to test for significance. *p-value* ≤ *0.05* is regarded as significant

Overall survival analysis was performed on patients with localized PDACs (group LOC). To visualize the prognostic effect of MP infiltration status in R0CRM– resected localized PDAC patients, overall survival (OS) analysis was performed on R0CRM– resected LOC patients stratified according to the infiltration status of the mesopancreas (*n* = 113, Table 2, bottom left). The median OS of the R0CRM– resected MP– LOC patients was with a median survival time of 21.0 months (95%CI: 10.47– 31.53 months) similar to the R0CRM– resected MP + LOC patients (median OS: 16.00 months 95%CI: 9.66 – 22.63 months) (*p* = *0.989*) (Fig. 3). Thus, the prognostic effect of margin negativity was not influenced by mesopancreatic fat infiltration.

## Discussion

Remarkably, refined histopathological assessment—implementing CRM—revealed that about 80% of pancreatic resections displayed microscopic tumor residues both at the dorsal and medial surgical margins, suggesting that a more thorough surgical approach and/or neoadjuvant therapy might lead to a better therapeutic outcome [22, 26]. In borderline resectable PDAC patients, vascular resections are often necessary to enhance the probability of achieving clear margins at the medial boundary, while mesopancreatic excision focuses on ensuring adequate clearance at both the ventral and dorsal margins. This procedure targets anatomical anchor points located between the celiac trunk and the superior mesenteric artery [17, 18].

The mesopancreas remains a controversially discussed area [16, 27–30]. Considering the embryologic origin of the pancreas and its secondary retroperitoneal nature, we should not doubt the existence of a mesopancreas [14, 25]. Since it was first mentioned in the contemporary literature of the early twenty-first century [15, 16, 25], the clinical significance of the mesopancreas remained poor. Total mesocolic and mesorectal excision have however received surgical acceptance because, amongst others, the peri- colonic and rectal fascial system [12, 31] enabled a reproducible technique. A peripancreatic fascial system is present but until now poorly studied [13, 14, 25]. After the first results of the pancreatic LEEDS protocol [1, 2] with the resulted incline in R1 resection rate [5] as well as the super survival stratification with the 1 mm rule (R0CRM-) [26], the surgical view on the degree of radical pancreatic surgery needs to change. The mesopancreas and its excision could be an answer to improve local tumor control. The current data in the known literature provides enough evidence that tumor extensions into the mesopancreas were frequently involved. We have already quantified the infiltration status of the mesopancreatic fat in a series of consecutively treated PDAC patients [18]. We further revealed in a small series of neoadjuvant treated borderline resectable PDAC patients the lower rate of mesopancreatic infiltration status in highly responsive cases [20].

We do not know the risk of mesopancreatic infiltration in localized PDACs, who are upfront resected and regarded to have a low-risk oncological profile. Primary resectable patients represent a subgroup of patients in whom the risk of vascular infiltration to the portomesenteric system or arterial vasculate is marginal. Since we used the postoperative histopathological outcome of the medial tumor extensions, we were able to remove patients who have falsely received upfront resection according to current preoperative staging standards [6]. In our opinion this makes our methodology unique, since we eliminated radiographic confounder and prediction bias.

The study has some limitations, which must be considered. First, the retrospective and unicentric nature of the study could have led to an unknown bias – yet a large and homogenously operated collective cannot be analyzed in a different fashion. Second, we did not assess preoperative CA-19–9 values or the clinical performance status of the patients. According to the ABC-scheme in current international guidelines, these variables, as well as anatomical factors, are being considered for resectability stratification [6, 32]. Nonetheless, the anatomical relation of the PDAC to major vessels remains the leading preoperative stratification variable [6]. One major criticism is that patients with a borderline resectable disease have not received neoadjuvant treatment, which represents the current gold standard. A correlation of mesopancreatic infiltration status between our subgroups do not meet current standards. However, the aim of this study was to provide results on the infiltration status of the mesopancreas in localized disease who currently not enter neoadjuvant therapy. Such patients have been identified and these results are below discussed. To the best of our knowledge, similar data is not available in the literature.

Mesopancreatic fat infiltration was notably lower among patients with localized PDACs; however, the mesopancreas remained affected in more than 69% of the cases, highlighting a remained risk profile in this group. Notably, in those with localized disease, positive mesopancreatic involvement was significantly associated with a higher rate of R1 resections at the dorsal margin (81% R0CRM- resections in MP-negative patients vs. 63% in MP-positive patients, *p* = *0.019*). The MP infiltration status did not cause an adverse survival outcome if margin negativity (R0CRM–) was secured, presuming that a positive infiltration into the mesopancreas is rather related to tumor topography instead of aggressive tumor biology.

In our opinion these important findings clearly show underestimated tumor extensions which might question current resectability criteria [6]. The current stratification models focus on the vascular involvement with the PDAC. From an oncological point of view this is important since those patients are at an increased risk for systemic disease. However successful local tumor control (R0CRM- resection at every margin) with a resulted decreased risk of local recurrence [18] is similarly necessary to achieve long term survival. The dorsal margin, the mesopancreas, must be included during risk assessment to successfully integrate all areas of tumor extension and provide the possibility to achieve both local and systemic tumor control. Since the infiltration status of the mesopancreas significantly affects the dorsal resection margin status in primary resectable PDAC patients, we must discuss if these patients truly harbor a localized disease. Further studies are imminent to study the effect of neoadjuvant therapy in’’primary-resectable’’ PDAC patients and the downsizing effect on the mesopancreatic infiltration status with the resection margin status.

During staging and resectability stratification the area of the mesopancreas is not assessed. As we and others have previously reported, patients with an increased fat stranding in the mesopancreas on preoperative CT scans were found to have a higher likelihood of mesopancreatic fat infiltration and were less prone to receive a curative R0CRM- resection [19, 21]. Furthermore, tumor response on CT following neoadjuvant therapy was associated with a reduced risk of mesopancreatic fat infiltration [20]. Postoperative histopathological staging includes all relevant resection margins, but the preoperative focus remains on one area. Including the radiographic assessment of the mesopancreas in preoperative staging is possible and could enable a comprehensive evaluation of tumor extension and enhance current therapeutic management and staging.

## Conclusion

Infiltration to the mesopancreas in localized PDAC who currently enter upfront resection remained high. R0CRM– resection status was mostly prevalent in localized PDAC patients without invasion of the mesopancreas, making these patients candidates for a safe resection and eligible for upfront surgery. Current resectability criteria have to be discussed Further studies are warranted to study the downsizing effects on mesopancreatic infiltration by neoadjuvant treatment in primary resectable and borderline resectable PDAC patients.

## Data Availability

The datasets used and/or analyzed during the current study are available from the corresponding author on reasonable request.

## Abbreviations

ADV: Advanced disease (positive vascular groove)
CHA: Common hepatic artery
CI: Confidence interval
CRM: Circumferential resection margin
GDA: Gastroduodenal artery
HR: Hazard ratio
L: Lymphatic invasion
LEEPP: Leeds Pathology Protocol
LOC: Localized disease (negative vascular groove)
PD: Pancreatoduodenectomy
PDAC: Ductal adenocarcinoma of the pancreatic head
Pn: Perineural invasion
PV/SMV: Portal/superior mesenteric vein
RFS: Relapse free survival
SMA: Superior mesenteric artery
UICC: Union for international cancer control
V: Venous invasion
